# Robust Combined Binarization Method of Non-Uniformly Illuminated Document Images for Alphanumerical Character Recognition

**DOI:** 10.3390/s20102914

**Published:** 2020-05-21

**Authors:** Hubert Michalak, Krzysztof Okarma

**Affiliations:** Faculty of Electrical Engineering, West Pomeranian University of Technology in Szczecin, 70-313 Szczecin, Poland; michalak.hubert@zut.edu.pl

**Keywords:** image binarization, optical character recognition, document images, local thresholding, image pre-processing, natural images

## Abstract

Image binarization is one of the key operations decreasing the amount of information used in further analysis of image data, significantly influencing the final results. Although in some applications, where well illuminated images may be easily captured, ensuring a high contrast, even a simple global thresholding may be sufficient, there are some more challenging solutions, e.g., based on the analysis of natural images or assuming the presence of some quality degradations, such as in historical document images. Considering the variety of image binarization methods, as well as their different applications and types of images, one cannot expect a single universal thresholding method that would be the best solution for all images. Nevertheless, since one of the most common operations preceded by the binarization is the Optical Character Recognition (OCR), which may also be applied for non-uniformly illuminated images captured by camera sensors mounted in mobile phones, the development of even better binarization methods in view of the maximization of the OCR accuracy is still expected. Therefore, in this paper, the idea of the use of robust combined measures is presented, making it possible to bring together the advantages of various methods, including some recently proposed approaches based on entropy filtering and a multi-layered stack of regions. The experimental results, obtained for a dataset of 176 non-uniformly illuminated document images, referred to as the WEZUT OCR Dataset, confirm the validity and usefulness of the proposed approach, leading to a significant increase of the recognition accuracy.

## 1. Introduction

The increasing interest in machine and computer vision methods, recently observed in many areas of industry, is partially caused by the growing availability of relatively inexpensive high quality cameras and the rapid growth of the computational power of affordable devices for everyday use, such as mobile phones, tablets, or notebooks. Their popularity makes it possible to apply some image processing algorithms in many new areas related to automation, robotics, intelligent transportation systems, non-destructive testing and diagnostics, biomedical image analysis, and even agriculture. Some methods, previously applied, e.g., for visual navigation in mobile robotics, may be successfully adopted for new areas, such as automotive solutions, e.g., Advanced Driver-Assistance Systems (ADAS). Nevertheless, such extensions of previously developed methods are not always straightforward, since the analysis of natural images may be much more challenging in comparison to those acquired in fully controlled lighting conditions.

One of the dynamically growing areas of the applications of video technologies based on the use of camera sensors is related to the utilization of Optical Character Recognition (OCR) systems. Some of them include: document image analysis, recognition of the QR codes from natural images [1,2], as well as automatic scanning and digitization of books [3], where additional infrared cameras may also be applied, e.g., supporting the straightening process for the scanned pages. Considering the wide application possibilities of binary image analysis for shape recognition, also in embedded systems with limited computational power and a relatively small amount of memory, a natural direction seems to be their utilization in mobile devices. Since modern smartphones are usually equipped with multi-core processors, some parallel image processing methods may be of great interest as well.

As images acquired by vision sensors in cameras are usually full color photographs, which may be easily converted into grayscale images (if they are not acquired by monochrome sensors directly), the next relevant pre-processing step is their conversion into binary images, significantly decreasing the amount of data used in further shape analysis and character recognition. Nevertheless, for the images captured in uncontrolled lighting conditions, the presence of shadows, local light reflections, illumination gradients, and other background distortions may lead to an irreversible loss of information during the image thresholding, causing many errors in character recognition. Hence, an appropriate binarization of such non-uniformly illuminated images is still a challenging task, similar to degraded historical document images containing many specific distortions.

To face this challenge, many various algorithms have been proposed during recent years, i.e., presented at the Document Image Binarization Competitions (DIBCO) organized during the two most relevant conferences in this field: the International Conference on Document Analysis and Recognition (ICDAR) [4] and the International Conference on Frontiers in Handwriting Recognition (ICFHR) [5]. All competitions have been held with the use of dedicated DIBCO datasets (available at: https://vc.ee.duth.gr/dibco2019/) containing degraded handwritten and machine-printed historical document images together with their binary “ground-truth” (GT) equivalents used for verification of the obtained binarization results.

Since there is no single binarization method that would be perfect for all applications for document images, some initial attempts at the combination of widely known approaches have been made [6], although verified for a relatively small number of test images from earlier DIBCO datasets. Another interesting recent idea is the development of some methods, which should be balanced between the processing time and obtained accuracy, presented during the ICDAR 2019 Time-Quality Document Binarization Competition [7]. Some approaches presented during this competition were also based on the combination of multiple methods, e.g., based on supervised machine learning, including texture features, with the use of the XGBoost classifier and additional morphological post-processing, as well as, e.g., a combination of the Niblack [8] and Wolf [9] methods. Nonetheless, such approaches typically do not focus on document images and OCR applications, considering image binarization as a more general task.

Some attempts at the combination of various methods, also using quite sophisticated approaches, have also been made for the images captured by portable cameras [10,11,12]. Some of the algorithms have been implemented in PhotoDoc [13], a software toolbox designed to process document images acquired with portable digital cameras integrated with the Tesseract OCR engine. A more comprehensive overview of the analysis methods of text documents acquired by cameras may be found in the survey paper [14].

Nevertheless, in view of potential parallelization of processing, an appropriate combination of some recently proposed binarization methods, also with some previously known algorithms, may lead to relatively fast and accurate results in terms of the OCR accuracy.

Although the most common approaches to the assessment of image binarization are based on the comparison of individual pixels [15,16], it should be noted that not all improperly classified pixels have the same influence on the final recognition results. Obviously, incorrectly classified background pixels located in the neighborhood of characters may be more troublesome than single isolated points in the background. Regardless of the presence of some pixel-based measures, such as, e.g., the pseudo-F-measure or Distance Reciprocal Distortion (DRD) [17], considering the distance of individual pixels from character strokes, their direct application would require not only the presence of the GT images, but also their precise matching with acquired photos. Hence, considering the final results of the character recognition, the assessment of thresholding methods considered in the paper is conducted by the calculation of the number of correctly and incorrectly recognized alphanumerical characters instead of single pixels.

One of the main goals of the conducted experiments is the verification of possible combinations of the recently proposed methods [18,19,20] with some other algorithms, without a priori training, therefore excluding some recently proposed deep learning approaches due to their memory and hardware requirements. To minimize the direct impact of camera parameters and properties on the characteristics of the obtained image and further processing steps, a Digital Single Lens Reflex (DSLR) camera Nikon N70 is used to acquire the images. The main contributions of the paper are the proposed idea of the combination of some recently proposed image binarization methods, particularly utilizing image entropy filtering and multi-layered stack of regions, based on pixel voting, with additional tuning of some parameters of the selected algorithms, as well as verification for the developed image dataset, containing 176 non-uniformly illuminated document images.

The rest of the paper contains an overview of the most popular image thresholding algorithms, including recently proposed ideas of image pre-processing with entropy filtering [18], background modeling with image resampling [19], and the use of a multi-layered stack of image regions [20], as well as the discussion of the proposed approach, followed by the presentation and analysis of the experimental results and final conclusions.

## 2. Overview of Image Binarization Algorithms

Image binarization has a relatively long history due to a constant need to decrease the amount of image data, caused earlier by the limitations of displays, the availability of memory, as well as processing speed. The simplest methods of global binarization of grayscale images are based on the choice of a single threshold for all pixels of the image. Instead of the simplest choice of 50% of the dynamic range, the Balanced Histogram Thresholding (BHT) method may be applied [21], where the threshold should be chosen in the lowest part of the histogram’s valley. However, this fast and simple method, initially developed for biomedical images, should be applied only for images with bi-modal histograms due to some problems with big tails in the histogram, being useless for unevenly illuminated document images. Kittler and Illingworth proposed an algorithm [22] minimizing the Bayes misclassification error expressed as the solution of the quadratic equation, assuming the normal distribution of the brightness levels for objects and background, further improved by Cho et al. [23] using the model distributions with corrected variance values.

Another global method, regarded as the most popular one for images with bi-model histograms, was proposed by Nobuyuki Otsu [24]. Its idea utilizes the maximization of inter-class variance equivalent to the minimization of the sum of two intra-class variances calculated for two groups of pixels, representing the foreground and background, respectively. A similar approach, although replacing the variance with the histogram’s entropy, was proposed by Kapur et al. [25]. Since both methods work properly only for uniformly illuminated images, their modifications utilizing the division of images into regions and combining the obtained local and global thresholds were also considered a few years ago [26].

A more formal analysis of the similarities and differences between some global thresholding methods for bi-modal histogram images, including the iterative selection method proposed by Ridler and Calvard [27], may be found in the paper [28]. Nevertheless, these methods do not perform well for natural images, where the bi-modality of the histogram cannot be ensured. A similar problem may be found applying some other methods developed for binarization of images with unimodal histograms [29,30], which are not typical for document images as well.

An obvious solution of these problems is the use of adaptive binarization methods, where the threshold values are determined locally for each pixel, depending on the local parameters, such as average brightness or local variance. In some cases, semi-adaptive versions of global thresholding may be applied as the region based approaches, where different thresholds may be set for various image fragments. One of exemplary extensions of the classical Otsu’s method, referred to as AdOtsu, was proposed by Moghaddam and Cheriet [31], who postulated the use of the additional detection of line heights and stroke widths, as well as the multi-scale background estimation and removal.

The region based thresholding using Otsu’s method with Support Vector Machines (SVM) was proposed by Chou et al. [32], whereas another application of SVMs with local features was recently analyzed by Xiong et al. [33]. Some relatively fast region based approaches were proposed recently as well [34,35], leading finally to the idea of the multi-layered stack of regions [20].

Apart from the above-mentioned method proposed by Kapur et al. [25], some entropy based binarization methods may be distinguished as well. Some of them, although less popular than histogram based algorithms, utilize the histogram’s entropy [36,37], whereas some other approaches are based on the Tsallis entropy [38] or Shannon entropy with the classification of pixels into text, near-text, and non-text regions [39]. Some earlier algorithms, e.g., developed by Fan et al. [40], were based on the maximization of the 2D temporal entropy or minimization of the two-dimensional entropy [41]. Some more sophisticated ideas employ genetic methods [42] and cross-entropy for color image thresholding, as presented in a recent paper [43]. Another recent idea is the application of image entropy filtering for pre-processing of unevenly illuminated document images [18], which may be applied in conjunction with some other thresholding methods, leading to significant improvement, particularly for some simple methods, such as, e.g., Meanthresh, which is based just on the calculation of the mean intensity of the local neighborhood and setting it as the local threshold value.

Another simple local thresholding method using the midgray value, defined as the average of the minimum and the maximum intensity within the local window, was proposed by Bernsen [44]. Although this method may be considered as relatively old, its modification for blurred and unevenly lit QR codes has been proposed recently [45], based on its combination with the global Otsu’s method. A popular adaptive binarization method, available in the MATLAB environment as the adaptthresh function, was proposed by Bradley and Roth [46], who applied the integral image for the calculation of the local mean intensity of the neighborhood, as well as the local median and Gaussian weighted mean in its modified versions. A description of some other applications of integral images for adaptive thresholding may be found in the paper [47].

One of the most widely known extensions of the above simple methods, such as Meanthresh or Bernsen’s thresholding, was proposed by Niblack [8], who used the mean local intensity lowered by the local standard deviation multiplied by the constant parameter k = −0.2 as the local threshold. The default size of the local sliding window was 3 × 3 pixels, and therefore, the method was very sensitive to local distortions. A simple, but efficient modification of this algorithm, known as the NICK method, was proposed by Khurshid et al. [48] for brighter images with the additional correction by the average local intensity and the changed parameter k = −0.1. One of the most popular extensions of this approach was proposed by Sauvola and Pietikäinen [49], where the additional use of the dynamic range of the standard deviation was applied. The additional modifications of this approach were proposed by Wolf and Jolion [9], who used the normalization of contrast and average intensity, as well as by Feng and Tan [50], using the second larger local window for the computation of the local dynamic range of the standard deviation. The latter approach was relatively slow because of the application of additional median filtration with bilinear interpolation. A multi-scale extension of Sauvola’s method was proposed by Lazzara and Géraud [51], whereas the additional pre-processing with the use of the Wiener filter and background estimation was used by Gatos et al. [52], together with noise removal and additional post-processing operations.

Another algorithm, known as the Singh method [53], utilizes integral images for local mean and local mean deviation calculations to increase the speed of computations. One of the most recent methods based on Sauvola’s algorithm, referred to as ISauvola, was proposed in the paper [54], where the local image contrast was applied to adjust the method’s parameters automatically. Another modification of Sauvola’s method applied to QR codes with an adaptive window size based on lighting conditions was recently presented by He et al. [55], who used an adaptive window size partially inspired by Bernsen’s approach. Another recently proposed algorithm, inspired by Sauvola’s method, named WANafter the first name of one of its authors [56], focuses on low contrast document images, where the local mean values are replaced by so-called “maximum mean”, being in fact the average of the mean and maximum intensity values. Nevertheless, this approach was verified only for the H-DIBCO 2016 dataset, containing 14 handwritten images; hence, it might be less suitable for machine-printed document images and OCR applications.

Some other methods inspired by Niblack’s algorithm were also proposed by Kulyukin et al. [57] and by Samorodova and Samorodov [58]. The application of dynamic windows for Niblack’s and Sauvola’s methods was presented by Bataineh et al. [59], whereas Mysore et al. [60] developed a method useful for binarization of color document images based on the multi-scale mean-shift algorithm. A more detailed overview of adaptive binarization methods based on Niblack’s approach, as well as some others, may be found in some recent survey papers [61,62,63,64,65,66].

Some researchers developed many less popular binarization methods, which were usually relatively slow, and their universality was limited due to some assumptions related to necessary additional operations. For example, an algorithm described by Su et al. [67] utilized a combination of Canny edge filtering and an adaptive image contrast map, whereas Bag and Bhowmick [68] presented a multi-scale adaptive–interpolative method, dedicated for documents with faint characters. Another method based on Canny edge detection was presented by Howe [69], who combined it with the Laplacian operator and graph cut method, leading to an energy minimization approach. An interesting method based on background suppression, although appropriate mainly for uniformly lit document images, was developed by Lu et al. [70], whereas Erol et al. [71] used a generalized approach to background estimation and text localization based on morphological operations for documents acquired by camera sensors from mobile phones. The mathematical morphology was also used in the method presented by Okamoto et al. [72].

An algorithm utilizing median filtering for background estimation was recently proposed by Khitas et al. [73], whereas Otsu’s thresholding preceded by the use of curvelet transform was described by Wen et al. [74]. Alternatively, Mitianoudis and Papamarkos [75] presented the idea of using local features with Gaussian mixtures. The use of the non-local means method before the adaptive thresholding was examined by Chen and Wang [76], and the method known as Fast Algorithm for document Image Restoration (FAIR) utilizing rough text localization and likelihood estimation was presented by Lelore and Bouchara [77], who used the obtained super-resolution likelihood image as the input for a simple thresholding. The gradient based method for binarization of medical and document images proposed by Yazid and Arof [78] utilized edge detection with the Prewitt filter for the separation of weak and strong boundary points. However, the presented results were obtained using only the document images from the H-DIBCO 2012 dataset.

Some other recent ideas are the use of variational models [79], fast background estimation based on image resampling [19], as well as the application of independent thresholding of the RGB channels of historical document images [80] with the use of Otsu’s method. Nevertheless, the latter method requires the additional training of the decision making block with the use of synthetic images. Due to recent advances of deep learning, some attempts were also made [81,82]; although, such approaches needed relatively large training image datasets, and therefore, their application may be troublesome, especially for mobile devices working in uncontrolled lighting conditions. Another issue is related to their high memory requirements, as well as the necessity of using some modern GPUs, which may be troublesome, e.g., in embedded systems, as well as in some industrial applications.

Recently, some applications of the fuzzy approach to image thresholding were also investigated by Bogatzis and Papadopoulos [83,84], as well as the use of Structural Symmetric Pixels (SSP) proposed by Jia et al. [85,86] (the original implementation of the method available at: https://github.com/FuxiJia/DocumentBinarizationSSP). The idea of this method is based on the assumption that the local threshold should be estimated using only the pixels around strokes whose gradient magnitudes are relatively big and directions are opposite, instead of the whole region.

## 3. Proposed Method

Apart from the approaches presented during the recent ICDAR [87], some initial attempts at the use of multiple binarization methods were made by Chaki et al. [6], as well as Yoon et al. [88], although the presented results were obtained for a limited number of test images taken from earlier DIBCO datasets or captured images of vehicles’ license plates. The idea of the combination of various image binarization based on pixel voting presented in this paper was verified using the 176 non-uniformly illuminated document images containing various kinds of illumination gradients, as well as five common font families, also with additional style modifications (bold, italics, and both of them) and utilized the combination of recently proposed methods with some adaptive binarization algorithms proposed earlier, based on different assumptions. The verification of the obtained results was done with the use of three various OCR engines, calculating the F-measure and OCR accuracy for characters, as well as the Levenshtein distance between two strings, which was defined as the number of character operations needed to convert one string into another. All the images were the photographs of the printed documents containing the well-known Lorem ipsum text acquired in various lighting conditions.

Assuming the parallel execution of three, five, or seven various image binarization algorithms, some differences in the resulting images may be observed, particularly in background areas. Nevertheless, the most significant fragments of document images were located near the characters subjected to further text recognition. The main idea of the proposed method of the voting of pixels being the result of the applications of individual algorithms for the same image was in fact equivalent to the choice of the median value of the obtained binary results (ones and zeros) for the same pixel using three, five, or seven applied methods. Obviously, one might not expect satisfactory results for the use of three similar methods, such as, e.g., Niblack’s, Sauvola’s, and Wolf’s algorithms, but for the approaches based on various assumptions, some of the results may differ significantly, being complementary to each other.

The preliminary choice of binarization methods for combination was made analyzing the performance of individual measures for Bickley Diary, Nabuco (dataset available at: https://dib.cin.ufpe.br/), and individual DIBCO datasets, using the typically used measures based on the comparison of pixels (accuracy, F-measure, DRD, MPM, etc.) reported in some earlier papers. Since these datasets, typically used for general-purpose document image binarization evaluation, do not contain ground-truth text data, the OCR accuracy results calculated for our dataset were additionally used for this purpose. Having found the most appropriate combination of three methods, the two additional methods were added in the second stage only to the best combinations of three methods, and finally, the next two methods were added only to the best such obtained combinations of five methods. The choice of the most appropriate candidate algorithms for the combination was made essentially among the algorithms, which individually led to relatively high OCR accuracy.

Considering this, as well as the complexity of many candidate methods, the combination of two recently proposed algorithms, namely image entropy filtering followed by Otsu’s global thresholding described in the paper [18] and the multi-layered stack of regions using 16 layers [20], with NICK adaptive thresholding [48], was proposed. Each of these methods may be considered as relatively fast, in particular assuming potential parallel processing, and based on different operations, as shown in earlier papers.

The application of the stack of regions [20] was based on the calculation of the thresholds for image fragments, where the image was divided into blocks partially overlapping each other; hence, each pixel belonged to different regions shifted from each other according to the specified layer, and the final threshold was selected as the average of the threshold values obtained for all regions to which the pixel belonged for different layers. The local thresholds for each region were calculated in a simplified form as T=a·mean(X)−b, where mean(X) is the local average, and the values of the optimized parameters were a = 0.95 and b=−7, as presented in the paper [20].

The application of the image entropy filtering based method [18] was conducted in a few main steps. The initial operation was the calculation of the local entropy, which could be made using MATLAB’s entropyfilt function, assuming a 17×17 pixel neighborhood (obtained after the optimization experiments), followed by its negation for better readability. The obtained entropy map was normalized and initially thresholded using Otsu’s method to remove the background information partially. Such an obtained image with segmented text regions was considered as the mask for the background subjected to morphological dilation used to fill the gaps containing the individual characters. The minimum appropriate size of the structuring element was dependent on the font size, and for the images in the test dataset, a 20 × 20 pixel size was sufficient. Such achieved background estimation was subtracted from the original image, and the negative of the result was subjected to contrast increase and final binarization. Since the above steps caused the equalization of image illumination and the increase of its contrast, various thresholding algorithms may be applied in the last step. Nevertheless, the best results of the further OCR in combination with the other methods were obtained for Otsu’s global thresholding applied as the last step of this algorithm.

The algorithm described in the paper [19], used in some of the tested variants, was based on the assumption that a significant decrease of the image size, e.g., using MATLAB’s imresize function, caused the loss of text information, preventing mainly the background information, similar to (usually much slower) low-pass filtering. Hence, the combination of downsampling and upsampling using the same kernel may be applied for a fast background estimation. In this paper, the best results were obtained using the scale factor equal to 8 and bilinear interpolation. Such an obtained image was subtracted from the original, and further steps were similar to those used in the previous method: increase of contrast (using the coefficient 0.4), negation, and the final global thresholding using Otsu’s method as well. Although both methods were based on similar fundamentals, the results of background estimation using the entropy filtering and image resampling differed significantly; hence, both methods could be considered as complementary to each other.

The last of the methods applied in the proposed approach, known as NICK [48], named after the first letter of its authors’ names, was one of the modifications of Niblack’s thresholding, where the local threshold is determined as:

(1) $$T=m+k\cdot s=m+k\cdot \sqrt{B}\;,$$

where *m* is the local average value, k = −0.2 is a fixed parameter, *s* stands for the local standard deviation, and hence, *B* is the local variance.

The modifications behind the NICK method lead to the formula:

(2) $$T=m+k\cdot \sqrt{B+m^{2}}\;,$$

with the postulated values of the parameter k = −0.1 for the OCR applications. As stated in the paper [48], the application of this value of *k* left the characters “crispy and unbroken” for the price of the presence of some noisy pixels. The window size originally proposed in the paper [48] was 19×19 pixels; however, the suitable parameters depended on the image size, as well as the font size and may be adjusted for specific documents. Nevertheless, after experimental verification, the optimal choice for the testing dataset used in this paper was a 15×15 pixel window with the “original” Niblack’s parameter k = 0.2.

Since most of the OCR engines utilized their predefined thresholding methods, which were integrated into the pre-processing procedures, the input images should be binarized prior the use of the OCR software to prevent the impact of their “built-in” thresholding. The well-known commercial ABBYY FineReader uses the adaptive Bradley’s method, whereas the freeware Tesseract engine developed by Google after releasing its source code by HP company [89] employs the global Otsu binarization. In this case, forced prior thresholding replaces the internal default methods of the OCR software.

## 4. Discussion of the Results

The experimental verification of the proposed combined image binarization method for the OCR purposes should be conducted using a database of unevenly illuminated document images, for which the ground truth text data are known. Unfortunately, currently available image databases, such as the DIBCO [4], Bickley Diary [90], or Nabuco datasets [87], used for the performance analysis of image binarization methods contain usually a handwritten text (in some cases, also machine-printed) subjected to some distortions such as ink fading, the presence of some stains, or some other local distortions.

**Figure 1:**
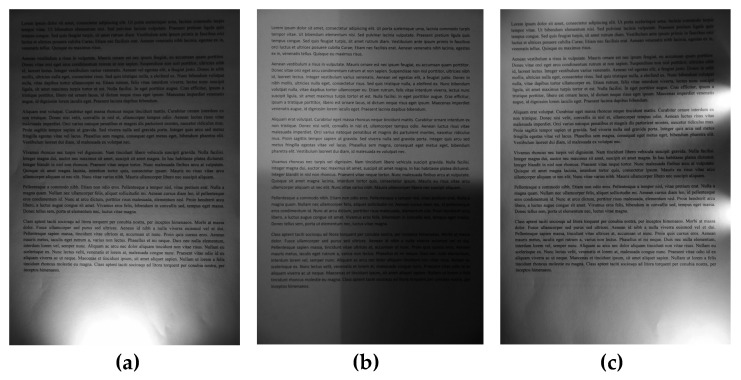
Three sample unevenly illuminated images from the dataset used in experiments. (**a**) with strongly illuminated bottom part; (**b**) with regular shadows; (**c**) with strongly illuminated right side.

Hence, a dedicated dataset containing 176 document images photographed by a Nikon N70 DSLR camera with a 70 mm focal length with the well-known Lorem ipsum text consisting of 563 words was developed with five font shapes, also with style modifications, and various types of non-uniform illuminations. Since the most popular font shapes were used, namely Arial, Times New Roman, Calibri, Verdana, and Courier, the obtained document images may be considered as representative for typical OCR applications. Three sample images from the dataset are shown in Figure 1. The whole dataset, referred to as the WEZUT OCR Dataset, has been made publicly available and may be accessed free of charge at http://okarma.zut.edu.pl/index.php?id=dataset&L=1.

For all images, several image binarization methods were applied, as well as their combinations based on the proposed pixel voting for 3, 5, and 7 methods. Such obtained images were treated as input data for three OCR engines: Tesseract (Version 4 with leptonica-1.76.0), MATLAB’s R2018a built-in OCR procedure (also originating from Tesseract), and GNU Ocrad (Version 0.27) based on a feature extraction method (software release available at: https://www.gnu.org/software/ocrad/). Since the availability of some other cloud solutions, usually paid, e.g., provided by Google or Amazon, may be limited in practical applications, we focused on two representative freeware OCR engines and MATLAB’s ocr function, which do not utilize any additional text operations related, e.g., to dictionary or semantic analysis.

Each result of the final text recognition was compared with ground truth data (the original Lorem ipsum text) using three measures: Levenshtein distance, interpreted as the minimum number of text changes (insertions, deletions, or substitutions of individual characters) needed to change a text string into another, as well as the F-measure and accuracy, typically used in classification tasks. The F-measure is defined as the harmonic mean of precision (true positives to all/true and false/positives ratio) and recall (ratio of true positives to the sum of true positives and false negatives), whereas accuracy may be calculated as the ratio of the sum of true positives and true negatives to all samples.

To verify the possibilities of the application of various combinations of different methods, the results of the proposed pixel voting approach were obtained using various methods. Nevertheless, only the best results are presented in the paper and compared with the use of individual thresholding methods. Most of the individual methods were implemented in MATLAB, although some of them partially utilized available codes provided in MATLAB Central File Exchange (Jan Motl) and GitHub (Doxa project by Brandon M. Petty). It is worth noting that the initial idea was the combination of three recently proposed approaches described in the papers [18,19,20]; hence, the first voting (Method No. #37 in Table 1 was used for these three algorithms (similar to the OR and AND operations shown as Methods #35 and #36 in Table 1). Nevertheless, during further experiments, better results were obtained replacing the resampling based method [19] with the NICK algorithm [48]. To illustrate the importance of an appropriate choice of individual methods for the voting procedure, some of the worse results (Methods #39–#41) are presented in Table 1, Table 2 and Table 3 as well. Further experiments with additional application of some other recent methods led to even better results.

A comparison of the results obtained for the whole dataset using Tesseract OCR is presented in Table 1, together with the rank positions for each of the methods. The overall rank was calculated using the rank positions achieved by each method according to three measures. Method #21 was the modification of Method #20 [18] with the use of the Monte Carlo method to speed up the calculations due to the decrease in the number of analyzed pixels. Nevertheless, applying the integral images in the methods referred to as #14–#20, it was possible to achieve even faster calculations. The results obtained for MATLAB’s built-in OCR and GNU Ocrad are presented in Table 2 and Table 3, respectively. A comparison of the processing time, relative to Otsu’s method, is shown in Table 4. The reference time obtained for Otsu’s method using a computer with Core i7-4810MQ processor (four cores/eight threads), 16GB of RAM, and an SSD disk was 1.77 ms.

Analyzing the results provided in Table 1, Table 2 and Table 3, it may be clearly observed that the best results were achieved using the Tesseract OCR, and the results obtained for the two remaining OCR programs should be considered as supplementary. Particularly poor results could be observed for the GNU Ocrad software. Among the various combinations based on voting, most of them achieved much better results than individual binarization methods regardless of the applied OCR engine, proving the advantages of the proposed approach. Nevertheless, considering the best results, it is worth noting that the use of only three methods (referred to as #58 in Table 1) provided the best F-measure and accuracy and the second results in terms of Levenshtein distance being better even in comparison with the voting approach with the use of five or seven individual algorithms. The Levenshtein distance achieved by this proposed method was only slightly worse than the result of pixel voting using seven algorithms (referred to as #61). Considering the worse OCR engines, some other combinations led to better results, especially for GNU Ocrad, where the application of seven methods referred to as #61 was not listed even in the top 10 methods. Therefore, the final aggregated rank positions for all three OCR engines, together with the relative computation time normalized according to Otsu’s thresholding, are presented in Table 4.

Although not all the results of the tested combinations of various methods are reported in Table 1, Table 2, Table 3 and Table 4, it is worth noting that the most successful combinations, leading to the best aggregated rank positions presented in Table 4, contained one of the variants of the multi-layered stack of regions (#20) or the resampling method (#19), as well as an entropy based method (#27). Therefore, the possibilities of the application of these recent approaches in combination with some other algorithms were confirmed. Considering additionally the processing time, a reasonable choice might also be the combination of Methods #22 and #27 with the recent ISauvola algorithm (#34), listed as #53, providing very good results for each of the tested OCR engines in view of Levenshtein distance.

**Figure 2:**
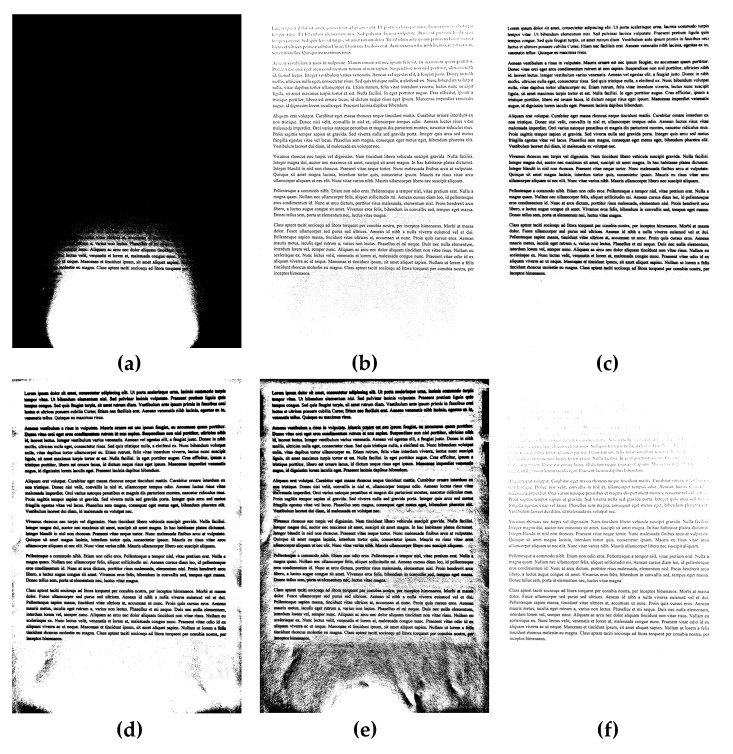

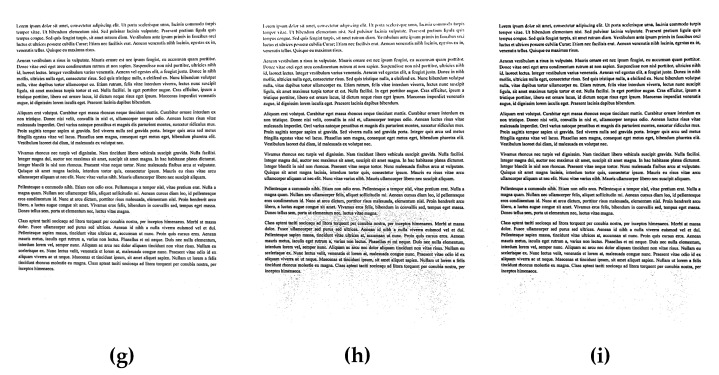
Binarization results obtained for a sample unevenly illuminated image from the dataset used in the experiments shown in Figure 1a for various methods: (**a**) Otsu, (**b**) Niblack, (**c**) Sauvola, (**d**) Bradley (mean), (**e**) Bernsen, (**f**) Meanthresh, (**g**) NICK, (**h**) stack of regions (16 layers), and (**i**) proposed (#51).

**Figure 3:**
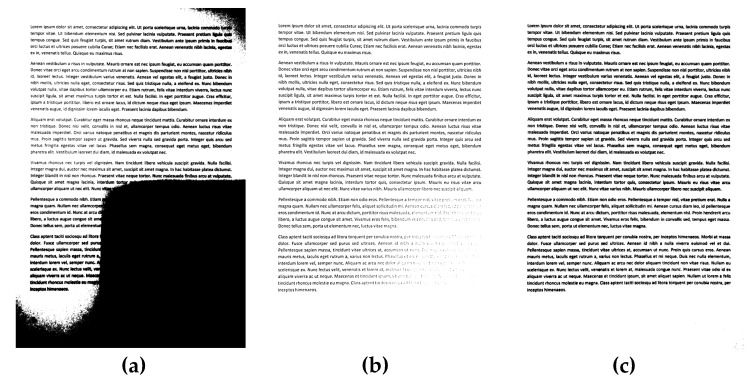

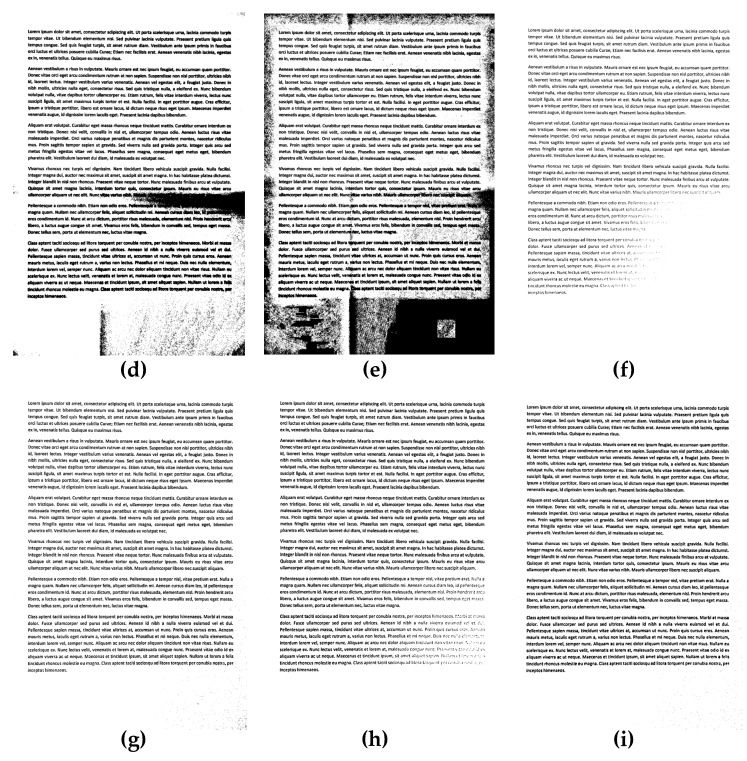
Binarization results obtained for a sample unevenly illuminated image from the dataset used in the experiments shown in Figure 1b for various methods: (**a**) Otsu, (**b**) Niblack, (**c**) Sauvola, (**d**) Bradley (mean), (**e**) Bernsen, (**f**) Meanthresh, (**g**) NICK, (**h**) stack of regions (16 layers), and (**i**) proposed (#51).

**Figure 4:**
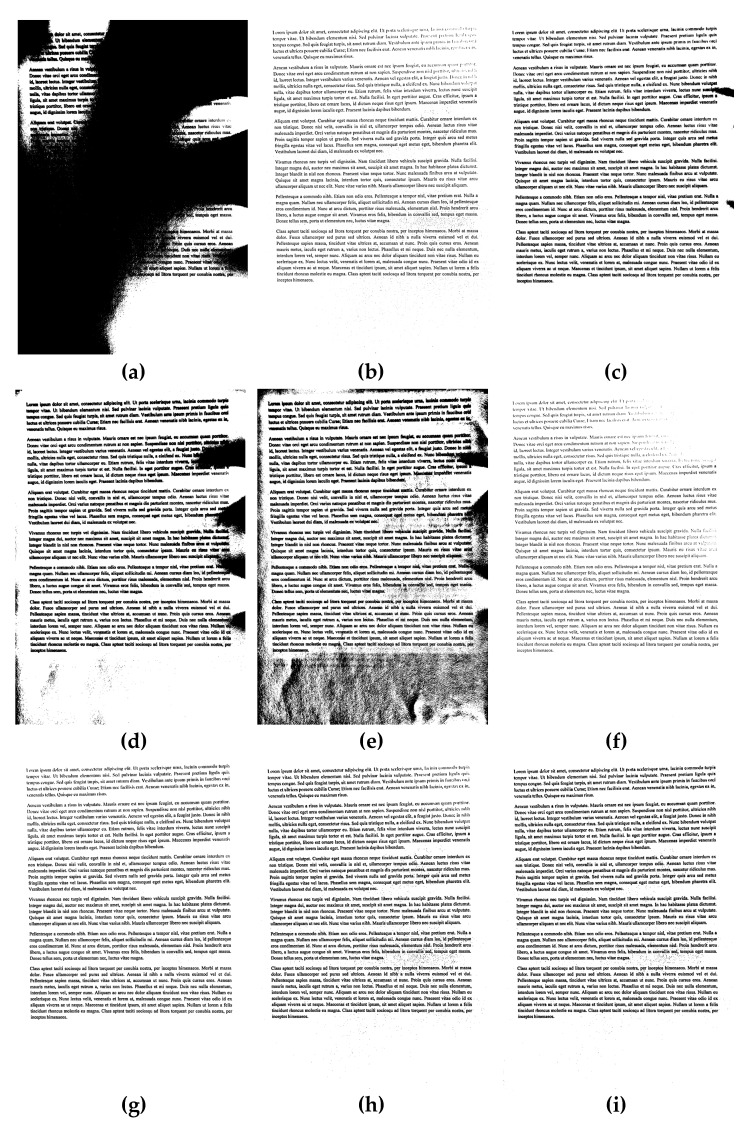
Binarization results obtained for a sample unevenly illuminated image from the dataset used in experiments shown in Figure 1c for various methods: (**a**) Otsu, (**b**) Niblack, (**c**) Sauvola, (**d**) Bradley (mean), (**e**) Bernsen, (**f**) Meanthresh, (**g**) NICK, (**h**) stack of regions (16 layers), and (**i**) proposed (#51).

Exemplary results of the binarization of sample documents from the dataset used in experiments are presented in Figure 2, Figure 3 and Figure 4, where significant differences between some methods may be easily noticed, as well as the relatively high quality of binary images obtained using the proposed approach.

## 5. Concluding Remarks

Binarization of non-uniformly illuminated images acquired by camera sensors, especially mounted in mobile devices, in unknown lighting conditions is still a challenging task. Considering the potential applications of the real-time analysis of binary images captured by vision sensors, not only directly related to OCR applications, but also, e.g., to mobile robotics or recognition of the QR codes from natural images, the proposed approach may be an interesting idea providing a reasonable accuracy for various types of illuminations.

The presented experimental results may be extended during future research also by the analysis of the potential applicability of the proposed methods and their combinations for automatic text recognition systems for even more challenging images, e.g., with metallic plates with embossed serial numbers. Another direction for further research may be the investigation of the potential applications of some fuzzy methods [83,84], which may be useful, e.g., for a combination of an even number of algorithms, as well as the use of different weights for each combined method.

## Figures and Tables

**Table 1:** Comparison of the average F-measure, Levenshtein distance, and Optical Character Recognition (OCR) accuracy values obtained for various binarization methods using the Tesseract OCR engine for 176 document images (three best results shown in bold format).

#	Binarization Method	OCR Measure						Overall Rank
		F-Measure	Rank	Levenshtein Distance	Rank	Accuracy	Rank	
1	Otsu [24]	0.6808	60	1469.88	60	0.5179	60	60
2	Chou [32]	0.8032	57	944.68	58	0.6575	57	57
3	Kittler [22]	0.6173	61	1889.86	61	0.3911	61	61
4	Niblack [8]	0.8838	48	243.39	47	0.7906	48	48
5	Sauvola [49]	0.9428	27	96.79	35	0.8955	27	28
6	Wolf [9]	0.9342	33	142.43	41	0.8800	30	36
7	Bradley (mean) [46]	0.9019	43	245.98	48	0.8217	43	45
8	Bradley (Gaussian) [46]	0.8490	51	557.98	54	0.7319	52	51
9	Feng [50]	0.7438	59	950.16	59	0.5908	59	59
10	Bernsen [44]	0.7673	58	724.68	57	0.6104	58	58
11	Meanthresh	0.8203	55	464.19	52	0.6885	55	54
12	NICK [48]	0.9551	24	43.20	25	0.9144	25	25
13	Wellner [91]	0.9134	40	275.10	50	0.8450	40	42
14	Region (1 layer) [20]	0.8858	46	174.98	42	0.7956	45	44
15	Region (2 layers) [20]	0.9236	38	105.19	36	0.8588	38	38
16	Region (4 layers) [20]	0.9344	31	92.14	31	0.8774	32	30
17	Region (6 layers) [20]	0.9359	30	93.24	32	0.8798	31	29
18	Region (8 layers) [20]	0.9341	34	88.88	29	0.8769	35	33
19	Region (12 layers) [20]	0.9343	32	93.33	33	0.8771	34	34
20	Region (16 layers) [20]	0.9339	35	90.65	30	0.8767	36	35
21	Region (16 layers + MC) [20]	0.9079	42	117.16	39	0.8315	42	41
22	Resampling [19]	0.9557	22	37.13	24	0.9156	23	23
23	Entropy + Otsu [18]	0.8418	53	618.51	56	0.7291	55	54
24	Entropy + Niblack [18]	0.8086	56	491.88	53	0.6758	56	56
25	Entropy + Bradley(Mean) [18]	0.9115	41	94.08	34	0.8405	41	39
26	Entropy + Bradley(Gauss) [18]	0.8908	44	188.71	43	0.8057	44	43
27	Entropy + Meanthresh [18]	0.9404	16	46.93	14	0.8899	17	15
28	SSP [85,86]	0.9402	28	111.99	27	0.8915	29	27
29	Gatos [52]	0.6808	49	1469.88	49	0.5179	49	50
30	Su [67]	0.9332	36	62.21	28	0.9772	33	32
31	Singh [53]	0.8945	25	245.57	23	0.8046	24	24
32	Bataineh [59]	0.3905	52	2578.68	51	0.1860	54	51
33	WAN [56]	0.9504	50	45.39	44	0.9080	50	49
34	ISauvola [54]	0.9459	26	80.53	26	0.8955	26	26
35	OR (#20,#22,#23)	0.9294	37	110.91	37	0.8698	37	37
36	AND (#20,#22,#23)	0.8408	54	615.75	55	0.7337	51	53
37	Voting (#20,#22,#23)	0.9576	18	30.44	17	0.9192	18	19
38	Voting (#5,#12,#22)	0.9585	16	31.35	22	0.9207	16	20
39	Voting (#4,#7,#11)	0.8863	45	236.19	45	0.7950	46	46
40	Voting (#4,#11,#22)	0.8844	47	238.95	46	0.7915	47	47
41	Voting (#7,#20,#23)	0.9206	39	141.19	40	0.8544	39	40
42	Voting (#7,#12,#20,#22,#23)	0.9568	20	29.05	12	0.9177	20	18
43	Voting (#12,#20,#23)	0.9617	8	26.82	8	0.9263	8	7
44	Voting (#12,#22,#27)	0.9586	15	30.88	19	0.9208	15	17
45	Voting (#12,#18,#20,#22,#27)	0.9576	19	31.04	21	0.9188	19	21
46	Voting (#5,#6,#12,#18,#20,#22,#27)	0.9617	7	27.11	9	0.9264	7	6
47	Voting (#16,#22,#23)	0.9556	23	30.93	20	0.9156	22	22
48	Voting (#12,#16,#23)	0.9605	10	27.95	10	0.9243	10	11
49	Voting (#7,#12,#16,#22,#23)	0.9580	17	29.52	13	0.9200	17	16
50	Voting (#20, #23, #34)	**0.9630**	3	26.39	7	**0.9289**	3	3
51	Voting (#20, #27, #31)	0.9602	12	23.31	5	**0.9289**	3	4
52	Voting (#20, #23, #28)	0.9623	6	25.52	6	0.9238	12	7
53	Voting (#22, #27, #34)	0.9597	13	**22.43**	3	0.9277	6	5
54	Voting (#22, #23, #31)	0.9560	21	28.60	11	0.9229	14	15
55	Voting (#22, #27, #28)	0.9630	4	23.11	4	0.9168	21	10
56	Voting (#28, #31, #34)	0.9597	14	30.82	18	0.9232	13	14
57	Voting (#20, #31, #34)	0.9603	11	30.44	16	0.9243	11	13
58	**Voting (#12, #28, #34)**	**0.9660**	1	**20.44**	2	**0.9346**	1	1
59	Voting (#22, #31, #34)	0.9611	9	30.02	15	0.9258	9	12
60	Voting (#4, #7, #28, #31, #34)	0.9626	5	29.88	14	0.9285	5	7
61	Voting (#12, #20, #22, #23, #28, #31, #34)	**0.9653**	2	**18.51**	1	**0.9333**	2	2

**Table 2:** Comparison of the average F-measure, Levenshtein distance, and OCR accuracy values obtained for various binarization methods using MATLAB’s built-in OCR engine for 176 document images (three best results shown in bold format).

#	Binarization Method	OCR Measure						Overall Rank
		F-Measure	Rank	Levenshtein Distance	Rank	Accuracy	Rank	
1	Otsu [24]	0.6306	60	1618.53	60	0.4368	60	60
2	Chou [32]	0.7351	54	1097.47	57	0.5495	55	56
3	Kittler [22]	0.5799	61	2027.23	61	0.3234	61	61
4	Niblack [8]	0.7395	52	455.53	45	0.5787	51	50
5	Sauvola [49]	0.8672	27	267.34	34	0.7655	28	29
6	Wolf [9]	0.8512	30	312.68	39	0.7433	30	31
7	Bradley (mean) [46]	0.8008	41	549.89	49	0.6554	41	43
8	Bradley (Gaussian) [46]	0.7554	47	856.21	55	0.5819	50	51
9	Feng [50]	0.6607	58	1041.55	56	0.4683	57	57
10	Bernsen [44]	0.6640	57	1194.11	58	0.4533	59	58
11	Meanthresh	0.7039	56	663.48	51	0.5212	56	55
12	NICK [48]	0.8589	29	208.87	26	0.7593	29	28
13	Wellner [91]	0.8268	32	470.24	46	0.6985	32	38
14	Region (1 layer) [20]	0.7136	55	455.26	44	0.5515	54	52
15	Region (2 layers) [20]	0.7852	43	286.34	37	0.6499	42	41
16	Region (4 layers) [20]	0.8059	38	249.19	33	0.6790	38	37
17	Region (6 layers) [20]	0.8095	37	249.01	32	0.6841	37	35
18	Region (8 layers) [20]	0.8151	35	239.34	29	0.6919	35	31
19	Region (12 layers) [20]	0.8141	36	241.31	30	0.6908	36	33
20	Region (16 layers) [20]	0.8160	34	242.50	31	0.6932	33	30
21	Region (16 layers + MC) [20]	0.7819	44	305.28	38	0.6429	43	42
22	Resampling [19]	0.8655	28	159.55	18	0.7677	27	26
23	Entropy + Otsu [18]	0.7734	46	786.19	54	0.6161	46	49
24	Entropy + Niblack [18]	0.6372	59	1211.35	59	0.4600	58	59
25	Entropy + Bradley(Mean) [18]	0.8212	33	363.03	41	0.6929	34	36
26	Entropy + Bradley(Gauss) [18]	0.7869	42	525.62	48	0.6398	44	44
27	Entropy + Meanthresh [18]	0.8790	21	149.66	15	0.7879	22	21
28	SSP [85,86]	0.8766	26	235.88	28	0.7802	26	27
29	Gatos [52]	0.7544	48	477.35	47	0.5936	47	46
30	Su [67]	0.8053	39	283.09	35	0.6763	39	39
31	Singh [53]	0.8779	23	185.99	24	0.7822	25	25
32	Bataineh [59]	0.8779	53	185.99	50	0.7822	53	54
33	WAN [56]	0.7461	50	742.53	52	0.5757	52	53
34	ISauvola [54]	0.8835	14	216.48	27	0.7890	20	23
35	OR (#20,#22,#23)	0.8049	40	285.87	36	0.6759	40	40
36	AND (#20,#22,#23)	0.7787	45	765.26	53	0.6269	45	47
37	Voting (#20,#22,#23)	0.8799	18	136.13	9	0.7899	18	14
38	Voting (#5,#12,#22)	0.8767	25	150.70	16	0.7854	24	24
39	Voting (#4,#7,#11)	0.7467	49	437.98	42	0.5887	48	45
40	Voting (#4,#11,#22)	0.7442	51	442.27	43	0.5840	49	47
41	Voting (#7,#20,#23)	0.8310	31	359.44	40	0.7067	31	33
42	Voting (#7,#12,#20,#22,#23)	0.8847	13	134.78	8	0.7977	11	8
43	Voting (#12,#20,#23)	0.8810	17	138.27	11	0.7924	15	12
44	Voting (#12,#22,#27)	0.8792	20	145.94	13	0.7888	21	20
45	Voting (#12,#18,#20,#22,#27)	0.8788	22	130.88	7	0.7892	19	18
46	Voting (#5,#6,#12,#18,#20,#22,#27)	0.8900	8	124.80	4	0.8064	7	5
47	Voting (#16,#22,#23)	0.8798	19	138.04	10	0.7902	17	16
48	Voting (#12,#16,#23)	0.8778	24	139.63	12	0.7868	23	22
49	Voting (#7,#12,#16,#22,#23)	0.8835	15	129.94	6	0.7953	13	10
50	Voting (#20, #23, #34)	0.8966	6	164.73	19	0.8112	6	7
51	Voting (#20, #27, #31)	**0.8993**	2	**118.30**	3	**0.8185**	2	1
52	Voting (#20, #23, #28)	0.8882	10	148.10	14	0.8002	9	9
53	Voting (#22, #27, #34)	0.8966	5	**116.29**	2	0.8141	5	4
54	Voting (#22, #23, #31)	0.8825	16	173.11	20	0.7905	16	19
55	Voting (#22, #27, #28)	**0.8983**	3	**114.48**	1	**0.8178**	3	1
56	Voting (#28, #31, #34)	0.8894	9	189.82	25	0.7991	10	13
57	Voting (#20, #31, #34)	0.8877	11	182.86	22	0.7971	12	14
58	Voting (#12, #28, #34)	0.8982	4	153.56	17	0.8163	4	6
59	Voting (#22, #31, #34)	0.8852	12	181.83	21	0.7932	14	17
60	Voting (#4, #7, #28, #31, #34)	0.8916	7	185.45	23	0.8025	8	11
61	Voting (#12, #20, #22, #23, #28, #31, #34)	**0.9014**	1	129.62	5	**0.8209**	1	1

**Table 3:** Comparison of the average F-measure, Levenshtein distance, and OCR accuracy values obtained for various binarization methods using GNU Ocrad for 176 document images (three best results shown in bold format).

#	Binarization Method	OCR Measure						Overall Rank
		F-Measure	Rank	Levenshtein Distance	Rank	Accuracy	Rank	
1	Otsu [24]	0.5622	60	2414.45	59	0.2231	60	59
2	Chou [32]	0.6013	56	1884.73	54	0.3316	56	56
3	Kittler [22]	0.5641	59	2487.22	60	0.2019	61	60
4	Niblack [8]	0.6639	43	953.84	36	0.4531	44	42
5	Sauvola [49]	0.7001	35	1136.26	44	0.4938	36	39
6	Wolf [9]	0.7068	32	1009.59	40	0.5083	34	36
7	Bradley (mean) [46]	0.6074	53	1786.23	52	0.3633	54	53
8	Bradley (Gaussian) [46]	0.5745	57	2151.78	58	0.2909	58	58
9	Feng [50]	0.6050	54	1943.19	55	0.3894	52	54
10	Bernsen [44]	0.5020	61	2969.76	61	0.2263	59	61
11	Meanthresh	0.6576	46	1075.15	42	0.4366	47	44
12	NICK [48]	0.7226	22	872.76	28	0.5362	21	21
13	Wellner [91]	0.6796	38	1214.53	46	0.4638	40	43
14	Region (1 layer) [20]	0.6183	51	1057.44	41	0.4038	50	47
15	Region (2 layers) [20]	0.6749	39	800.10	24	0.4780	38	34
16	Region (4 layers) [20]	0.6995	36	735.78	22	0.5098	33	30
17	Region (6 layers) [20]	0.7069	31	727.24	21	0.5198	28	25
18	Region (8 layers) [20]	0.7079	30	721.76	19	0.5210	27	24
19	Region (12 layers) [20]	0.7065	33	724.13	20	0.5195	29	28
20	Region (16 layers) [20]	0.7094	29	720.84	18	0.5237	24	21
21	Region (16 layers + MC) [20]	0.6661	41	824.98	26	0.4641	39	36
22	Resampling [19]	0.7331	18	690.13	16	0.5556	17	17
23	Entropy + Otsu [18]	0.6422	49	1420.09	49	0.4247	49	50
24	Entropy + Niblack [18]	0.6615	45	1962.02	56	0.4586	41	47
25	Entropy + Bradley(Mean) [18]	0.6247	50	1608.03	50	0.3917	51	51
26	Entropy + Bradley(Gauss) [18]	0.6142	52	1699.44	51	0.3740	53	52
27	Entropy + Meanthresh [18]	0.7558	8	573.63	9	0.5889	8	8
28	SSP [85,86]	0.7171	25	884.47	31	0.5225	25	27
29	Gatos [52]	0.6559	47	1178.52	45	0.4373	46	46
30	Su [67]	0.7020	34	814.40	25	0.5122	32	30
31	Singh [53]	0.7180	24	644.81	35	0.5219	26	29
32	Bataineh [59]	0.6041	55	1869.63	53	0.3609	55	55
33	WAN [56]	0.5695	58	2103.19	57	0.3109	57	57
34	ISauvola [54]	0.7109	28	1089.10	43	0.5068	35	36
35	OR (#20,#22,#23)	0.6879	37	883.67	30	0.4897	37	35
36	AND (#20,#22,#23)	0.6493	48	1390.91	48	0.4342	48	49
37	Voting (#20,#22,#23)	0.7565	7	558.05	6	0.5910	6	6
38	Voting (#5,#12,#22)	0.7422	14	675.09	14	0.5683	14	14
39	Voting (#4,#7,#11)	0.6665	40	937.72	33	0.4577	42	39
40	Voting (#4,#11,#22)	0.6648	42	965.40	37	0.4540	43	41
41	Voting (#7,#20,#23)	0.6636	44	1262.76	47	0.4492	45	45
42	Voting (#7,#12,#20,#22,#23)	0.7615	4	552.84	5	0.5980	4	4
43	Voting (#12,#20,#23)	0.7551	9	588.52	11	0.5883	9	10
44	Voting (#12,#22,#27)	0.7419	15	673.88	13	0.5679	15	15
45	Voting (#12,#18,#20,#22,#27)	0.7520	11	584.39	10	0.5846	11	11
46	Voting (#5,#6,#12,#18,#20,#22,#27)	0.7608	5	552.19	4	0.5968	5	5
47	Voting (#16,#22,#23)	0.7529	10	564.89	8	0.5853	10	9
48	Voting (#12,#16,#23)	0.7494	13	595.03	12	0.5799	12	12
49	Voting (#7,#12,#16,#22,#23)	0.7567	6	559.50	7	0.5906	7	7
50	Voting (#20, #23, #34)	0.7316	19	875.44	29	0.5412	19	19
51	Voting (#20, #27, #31)	**0.7673**	2	**530.68**	1	**0.6050**	2	2
52	Voting (#20, #23, #28)	0.7394	16	715.07	17	0.5587	16	16
53	Voting (#22, #27, #34)	**0.7679**	1	**531.49**	2	**0.6061**	1	1
54	Voting (#22, #23, #31)	0.7273	20	841.39	27	0.5386	20	19
55	Voting (#22, #27, #28)	**0.7661**	3	**537.62**	3	**0.6037**	3	3
56	Voting (#28, #31, #34)	0.7159	27	978.32	39	0.5174	31	33
57	Voting (#20, #31, #34)	0.7235	21	935.28	32	0.5287	22	23
58	Voting (#12, #28, #34)	0.7351	17	784.12	23	0.5504	18	18
59	Voting (#22, #31, #34)	0.7218	23	937.92	34	0.5268	23	25
60	Voting (#4, #7, #28, #31, #34)	0.7170	26	973.15	38	0.5189	30	32
61	Voting (#12, #20, #22, #23, #28, #31, #34)	0.7512	12	676.03	15	0.5761	13	13

**Table 4:** Comparison of the overall rank scores for 3 OCR engines and average computational time relative to Otsu’s method obtained for 176 document images.

#	Binarization Method	Final Aggregated Rank	Computation Time (Relative)	
1	Otsu [24]	60	1.00
2	Chou [32]	57	5.74
3	Kittler [22]	61	23.30
4	Niblack [8]	46	75.11
5	Sauvola [49]	33	73.73
6	Wolf [9]	36	76.36
7	Bradley (mean) [46]	47	19.62
8	Bradley (Gaussian) [46]	54	241.61
9	Feng [50]	58	215.20
10	Bernsen [44]	59	197.14
11	Meanthresh	51	39.93
12	NICK [48]	25	70.81
13	Wellner [91]	41	187.90
14	Region (1 layer) [20]	49	29.84
15	Region (2 layers) [20]	38	50.23
16	Region (4 layers) [20]	34	92.39
17	Region (6 layers) [20]	31	145.49
18	Region (8 layers) [20]	30	211.87
19	Region (12 layers) [20]	32	325.05
20	Region (16 layers) [20]	29	441.84
21	Region (16 layers + MC) [20]	40	1232.01
22	Resampling [19]	24	12.48
23	Entropy + Otsu [18]	51	664.87
24	Entropy + Niblack [18]	56	755.11
25	Entropy + Bradley(Mean) [18]	42	706.57
26	Entropy + Bradley(Gauss) [18]	45	932.92
27	Entropy + Meanthresh [18]	21	736.67
28	SSP [85,86]	27	4542.24
29	Gatos [52]	48	2413.68
30	Su [67]	35	6016.56
31	Singh [53]	26	59.78
32	Bataineh [59]	54	44.58
33	WAN [56]	53	400.98
34	ISauvola [54]	27	113.69
35	OR (#20,#22,#23)	37	1138.64
36	AND (#20,#22,#23)	50	1134.25
37	Voting (#20,#22,#23)	12	1136.87
38	Voting (#5,#12,#22)	22	159.17
39	Voting (#4,#7,#11)	43	137.30
40	Voting (#4,#11,#22)	44	130.64
41	Voting (#7,#20,#23)	39	1143.63
42	Voting (#7,#12,#20,#22,#23)	9	1224.28
43	Voting (#12,#20,#23)	7	1191.77
44	Voting (#12,#22,#27)	18	817.40
45	Voting (#12,#18,#20,#22,#27)	15	1455.67
46	Voting (#5,#6,#12,#18,#20,#22,#27)	4	1600.90
47	Voting (#16,#22,#23)	14	793.58
48	Voting (#12,#16,#23)	13	858.17
49	Voting (#7,#12,#16,#22,#23)	11	892.77
50	Voting (#20, #23, #34)	7	1249.60
51	Voting (#20, #27, #31)	1	1247.58
52	Voting (#20, #23, #28)	10	5662.15
53	Voting (#22, #27, #34)	2	887.61
54	Voting (#22, #23, #31)	19	801.04
55	Voting (#22, #27, #28)	3	5286.12
56	Voting (#28, #31, #34)	23	4584.69
57	Voting (#20, #31, #34)	15	745.37
58	Voting (#12, #28, #34)	6	4572.60
59	Voting (#22, #31, #34)	20	190.31
60	Voting (#4, #7, #28, #31, #34)	15	4656.10
61	Voting (#12, #20, #22, #23, #28, #31, #34)	4	5880.53

## Abbreviations

The following abbreviations are used in this manuscript:

ADAS	Advanced Driver-Assistance System
BHT	Balanced Histogram Thresholding
DIBCO	Document Image Binarization Competition
DSLR	Digital Single Lens Reflex
DRD	Distance Reciprocal Distortion
FAIR	Fast Algorithm for document Image Restoration
GPU	Graphics Processing Unit
GT	Ground Truth
H-DIBCO	Handwritten Document Image Binarization Competition
ICDAR	International Conference on Document Analysis and Recognition
ICFHR	International Conference on Frontiers in Handwriting Recognition
OCR	Optical Character Recognition
QR	Quick Response
SSD	Solid-State Drive
SSP	Structural Symmetric Pixels
SVM	Support Vector Machines
